# Ferroptosis Driver *SOCS1* and Suppressor *FTH1* Independently Correlate With M1 and M2 Macrophage Infiltration in Head and Neck Squamous Cell Carcinoma

**DOI:** 10.3389/fcell.2021.727762

**Published:** 2021-08-30

**Authors:** Zhang-Wei Hu, Yi-Hui Wen, Ren-Qiang Ma, Lin Chen, Xue-Lan Zeng, Wei-Ping Wen, Wei Sun

**Affiliations:** ^1^Department of Otolaryngology, The First Affiliated Hospital, Sun Yat-sen University, Guangzhou, China; ^2^Otorhinolaryngology Institute, Sun Yat-sen University, Guangzhou, China; ^3^Department of Otolaryngology, The Sixth Affiliated Hospital of Sun Yat-sen University, Guangzhou, China

**Keywords:** ferroptosis, suppressor of cytokine signaling-1, ferritin heavy chain, tumor microenvironment, immune therapy

## Abstract

**Objective:**

To investigate the role of ferroptosis, an iron-dependent form of non-apoptotic cell death, in the head and neck squamous cell carcinoma (HNSCC) immune microenvironment.

**Materials and Methods:**

A list of ferroptosis-related genes was obtained from the FerrDb database. Gene expression data were acquired from the cancer genome atlas (TCGA) and analyzed using the R language. Protein–protein interaction analysis was conducted using STRING and GeneMANIA. The correlations between gene expression levels and a patient’s survival were analyzed using GEPIA, the Kaplan–Meier estimate, and a multivariate Cox proportional hazards model. The expression results were verified using Oncomine and Human Protein Atlas data. We used the TIMER, GEPIA2, GEPIA2021, and TIMER2 databases to investigate the relationships between gene expression and infiltrating immune cells.

**Results:**

Analysis of differentially expressed genes (DEGs) identified nine each ferroptosis drivers and ferroptosis suppressors, among which four genes correlated with survival as follows: two drivers (*SOCS1*, *CDKN2A*) associated with better survival and two suppressors (*FTH1*, *CAV1*) associated with poorer survival. Multivariate Cox survival analysis identified *SOCS1* and *FTH1* as independent prognostic factors for HNSCC, and their higher expression levels were verified using Oncomine and HPA data. The results acquired using TIMER, GEPIA2, GEPIA2021, and TIMER2 data revealed that the driver *SOCS1* and the suppressor *FTH1* independently correlated with M1 and M2 macrophage infiltration.

**Conclusions:**

The ferroptosis driver *SOCS1* and suppressor *FTH1* are independent prognostic factors and that correlate with M1 and M2 macrophage infiltration in HNSCC. Targeting ferroptosis-immunomodulation may serve as a strategy to enhance the activity of immunotherapy.

## Introduction

Head and neck squamous cell carcinoma (HNSCC) is the sixth most common cancer worldwide (Siegel et al., 2020). In 2016, the United States Food and Drug Administration approved immunotherapy as second-line monotherapy for recurrent or metastatic HNSCC, and in 2019, as front-line treatment of inoperable HNSCC (Ferris et al., 2016; Seiwert et al., 2016; Burtness et al., 2019). Despite great progress in immunotherapy, only a small subset of patients with HNSCC respond to immune checkpoint inhibitors (Johnson et al., 2020), mainly because of the immunosuppressive microenvironment associated with tumor immunosuppressive cells, including tumor-associated macrophages (TAMs, also called M2d), regulatory T cells (Tregs), and other immunomodulatory cells (Watermann et al., 2021). Thus, decreasing the populations of such cells may serve as a strategy to improve the patients’ objective response rates to immunotherapy.

Ferroptosis, a term coined in 2012, is an iron-dependent form of non-apoptotic cell death (Dixon et al., 2012). On one hand, in tumor microenvironment ferroptosis seems to have a dual role in tumor promotion and suppression, depending on releasing damage-associated molecular patterns and activating immune response (Chen et al., 2021). On the other hand, ferroptosis is thought to have synergistic effects to suppress tumor growth in combination with other anti-tumor drugs, including immune checkpoint inhibitors (Roh et al., 2016; Wang et al., 2019). Currently, numerous studies focus on the role of ferroptosis in tumors, particularly in tumor cells and infiltrating antitumor immune cells (Wang et al., 2019; Chen et al., 2021). However, the role of ferroptosis in the functions of infiltrating immunosuppressive cells is unknown. We previously found that the expression of the ferroptosis suppressor gene *FTH1* positively correlated with macrophages in most solid tumors (Hu et al., 2021), indicating an important role for ferroptosis in regulating tumor immunity.

In the present study, we mined data acquired from FerrDb to comprehensively analyze the correlations between ferroptosis-related genes, including drivers and suppressors, as well as with tumor-infiltrating immune cells in HNSCC, with the goal of uncovering the potential role of ferroptosis in the immune response to HNSCC.

## Materials and Methods

### Data Sources

The expression levels of genes and clinical information regarding HNSCC were acquired from the cancer genome atlas (TCGA)^1^ through the UCSC Xena tool.^2^ The use of such open-access data did not require approval from the local ethics committee.

### Ferroptosis-Related Genes and Differentially Expressed Genes

A list of ferroptosis-related genes was obtained from the FerrDb database (Zhou and Bao, 2020)^3^ that includes 98 ferroptosis driver genes and 94 ferroptosis suppressor genes (Supplementary Table 1). Differentially expressed genes (DEGs) were analyzed using the empirical Bayes method with adjusted *p*-value (Benjamini and Hochberg FDR) through R (version 3.6.3), RStudio (version 1.2.5033), and the R LIMMA package (Linear Models for Microarray Data, version 3.42.2). Genes with log_2_FC absolute value higher than 1 and *q*-value lower than 0.01 were considered as DEGs. The correlations between genes were investigated using the Pearson’s correlation analysis.

### Protein–Protein Interaction Analysis

Protein–protein interactions (PPIs) were analyzed using the Search Tool for the Retrieval of Interacting Genes/Proteins dataset (STRING, version 11.0b)^4^ and the GeneMANIA dataset^5^ that provides a biological network integration method for predicting gene function.

### Univariate and Multivariate Analyses of Prognosis

Correlations between the expression levels of ferroptosis-related genes and overall survival (OS) were separately analyzed through Gene Expression Profiling Interactive Analysis (GEPIA)^6^ with 50% cutoff and the Kaplan–Meier Plotter^7^ with auto select best cutoff. Multivariate Cox proportional hazard model analysis was implemented using RStudio (version 1.2.5033) and the R SURVIVAL package (version 3.2-3).

### Oncomine Platform and the Human Protein Atlas

The mRNA levels of SOCS1 and FTH1 for the datasets GSE2379, GSE3524, GSE6791, and PMID14729608 were acquired from the Oncomine Platform.^8^ Through GraphPad Prism 7.0, the two-tailed unpaired *t*-test assuming equal variances was performed to analyze differences in gene expression. Moreover, protein immunohistochemistry for SOCS1 and FTH1 in normal and tumor tissues were obtained from the Human Protein Atlas (HPA).^9^

### Analysis of Immune Cell Infiltration

The correlations between gene expression and immune cell infiltration (B cells, CD8+ T cells, CD4+ T cells, macrophages, neutrophils, and dendritic cells) were investigated using the Tumor IMmune Estimation Resource (TIMER)^10^ tool. We used GEPIA2^11^ to perform pairwise gene correlation analysis. We estimated immune infiltration using the CIBERSORT, CIBERSORT-ABS, QUANTISEQ, MCP-COUNTER, XCELL, and EPIC algorithms through the TIMER2 resource.^12^ We investigated gene expression levels in various immune cells by CIBERSORT through the GEPIA2021.^13^

## Results

### Patients’ Baseline Characteristics

In total, the gene expression data and clinical characteristics of 528 HNSCC, 2 metastatic and 82 normal tissue samples from the TCGA database were included in the study. Patients’ baseline characteristics including age at diagnosis, sex, pathological T, pathological N, pathological M, and tumor stage are presented in Table 1.

**TABLE 1:** Baseline characteristics of the cancer genome atlas (TCGA) data.

Characteristic	n (%)	Characteristic	n(%)
Normal	82 (100%)	HNSCC	528 (100%)
**Age**		**Age**	
Mean	62.3	Mean	61.4
Range	26.2–87.7	Range	20.0–90.1
**Gender**		**Gender**	
Male	57 (69.5%)	Male	386 (73.1%)
Female	25 (30.5%)	Female	142 (26.9%)
Metastatic	2 (100%)	T	
Age		T1	49 (9.3%)
Mean	61.6	T2	140 (26.5%)
Range	55.7–67.5	T3	101 (19.1%)
Gender		T4	175 (33.1%)
Male	1 (50%)	Unknown	63 (11.9%)
Female	1 (50%)	N	
		N0	180 (34.1%)
		N+	248 (47.0%)
		Unknown	100 (18.9%)
		Stage	
		I	27 (5.1%)
		II	74 (14.0%)
		III	82 (15.5%)
		IV	270 (51.1%)
		Unknown	75 (14.2%)

### Gene Expression Screening and PPI Analysis

**FIGURE 1:**
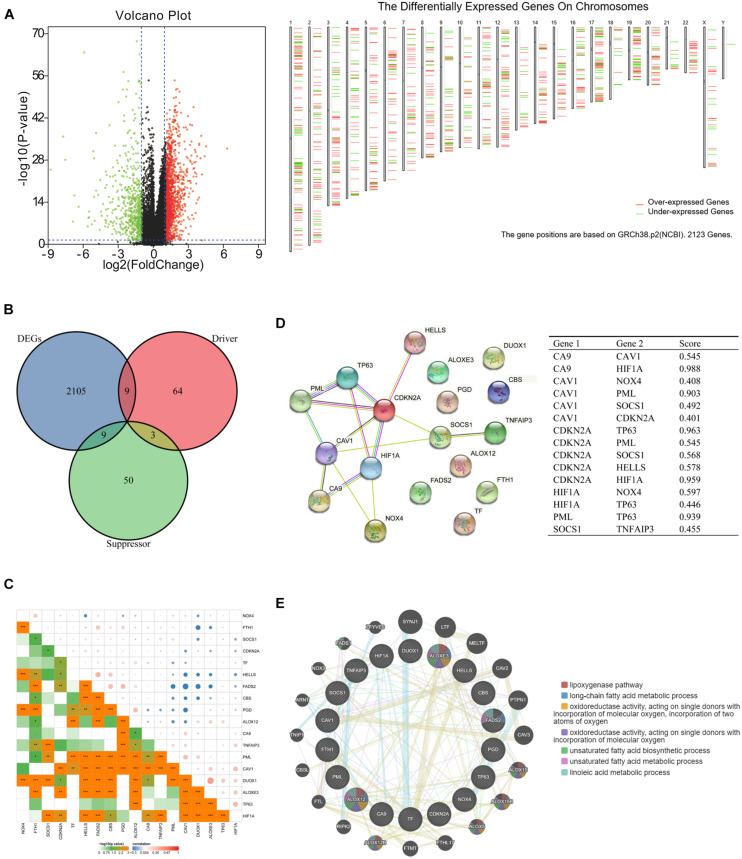
Ferroptosis-related differentially expressed genes (DEGs) and protein–protein interaction (PPI) analysis. **(A)** Volcano plot and chromosomal localizations of DEGs in head and neck squamous cell carcinoma (HNSCC) (“limma” analysis) **(B)** Venn diagram of common genes encoding ferroptosis drivers and suppressors that correlated with DEGs (R package analysis). **(C)** The correlation of expressions between each two genes of the 18 selected ferroptosis-related genes. **(D)** Annotation of ferroptosis-related differentially expressed proteins and their co-expression scores (STRING analysis). **(E)** PPI network of ferroptosis-related DEGs (GeneMANIA analysis), ALOXE3 and ALOXE12 were primarily related to the lipoxygenase pathway, while ALOXE3, ALOXE12, FADS2 were primarily related to the long-chain fatty acid metabolic process.

To explore the differentially expressed ferroptosis-related genes in HNSCC, mRNA expression was analyzed with the TCGA database. In total, we identified 2123 DEGs (Figure 1A), among which nine were ferroptosis-related drivers (*TNFAIP3*, *TF*, *SOCS1*, *PGD*, *NOX4*, *DUOX1*, *CDKN2A*, *ALOXE3*, *ALOX12*) and nine were ferroptosis-related suppressors (*TP63*, *PML*, *HIF1A*, *HELLS*, *FTH1*, *FADS2*, *CBS*, *CAV1*, *CA9*) (Figure 1B). Pearson correlation analysis was conducted to assess the correlations of expressions between each two genes of the above 18 ferroptosis related genes (Figure 1C). It showed that most of the gene expressions have statistical correlations and the detailed results were shown in the Supplementary Table 2. Then, we used STRING to analyze the PPI network, and the gene annotations and scores are listed in Figure 1D and Supplementary Table 3. The results of GeneMANIA revealed that ALOXE3 and ALOXE12 are primarily related to the lipoxygenase pathway, and ALOXE3, ALOXE12, FADS2 are primarily related to the long-chain fatty acid metabolic process (Figure 1E).

### *SOCS1* and *FTH1* Are Independent Prognostic Factors

**FIGURE 2:**
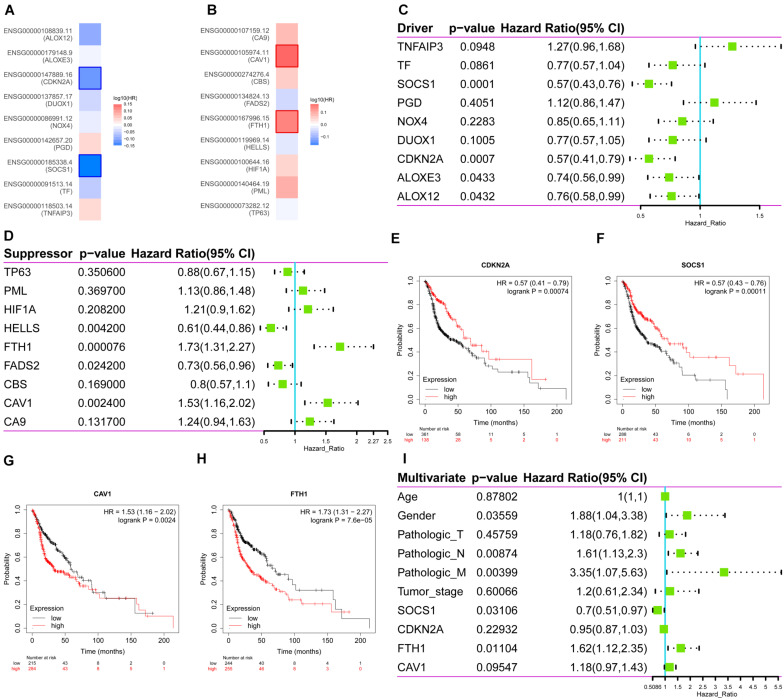
Survival analysis of the expression levels of the ferroptosis driver and suppressor genes in HNSCC. **(A)** The ferroptosis drivers *CDKN2A* and *SOCS1* correlated with longer overall survival (OS). **(B)** The ferroptosis suppressors *CAV1* and *FTH1* correlated with poorer OS. **(C–H)** Correlations between ferroptosis driver and suppressor genes and OS (Kaplan–Meier Plotter). **(I)** Multivariate Cox survival analysis showed that *SOCS1* (HR 0.7, 95%CI 0.51–0.97, *p* < 0.05) and *FTH1* (HR 1.62, 95%CI 1.12–2.35, *p* < 0.05) were independent prognostic factors for HNSCC.

Then, the correlations between ferroptosis-related DEGs and survival were analyzed, respectively through GEPIA (Figures 2A,B) and Kaplan–Meier Plotter (Figures 2C,D). Four common genes that correlated with survival were identified as follows: two drivers, *CDKN2A* (HR 0.57, 95%CI 0.41–0.79, *p* = 7.4 × 10^–4^) (Figure 2E); *SOCS1* (HR 0.57, 95%CI 0.43–0.76, *p* = 1.1 × 10^–4^) (Figure 2F); and two suppressors, *CAV1*, (HR 1.53, 95%CI 1.16–2.02, *p* = 2.4 × 10^–3^) (Figure 2G) and *FTH1* (HR 1.73, 95%CI 1.31–2.27, *p* = 7.6 × 10^–5^) (Figure 2H). Multivariate Cox survival analysis showed that *SOCS1* (HR 0.7, 95%CI 0.51–0.97, *p* < 0.05) and *FTH1* (HR 1.62, 95%CI 1.12–2.35, *p* < 0.05) were independent prognostic factors for HNSCC (likelihood ratio test *p* = 3 × 10^–6^, Wald test *p* = 5 × 10^–6^, score log-rank test *p* = 9 × 10^–9^) (Figure 2I).

### *SOCS1* and *FTH1* Are Expressed at Elevated Levels in HNSCC

**FIGURE 3:**
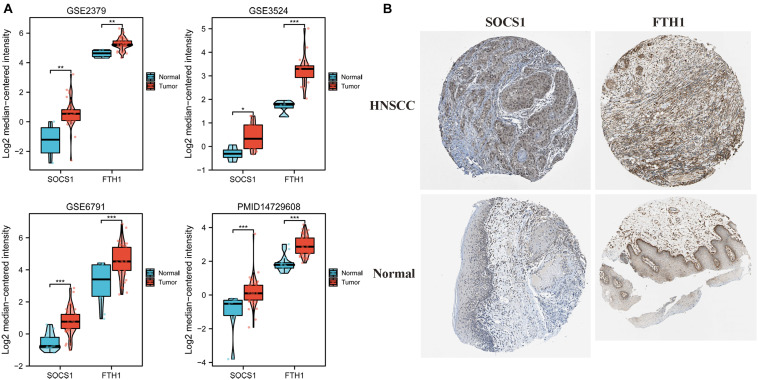
Analysis of *SOCS1* and *FTH1* gene expression using Oncomine and the HPA. **(A)** Validation of higher *SOCS1* and *FTH1* gene expression levels using the datasets GSE2379 (*SOCS1*, *t* = 3.569, *p* = 0.0010; *FTH1*, *t* = 3.168, *p* = 0.003), GSE3524 (*SOCS1*, *t* = 2.332, *p* = 0.0315; *FTH1*, *t* = 4.362, *p* = 0.0004), GSE6791 (*SOCS1*, *t* = 5.449, *p* < 0.0001; *FTH1*, *t* = 4.153, *p* = 0.0001), and PMID14729608 (*SOCS1*, *t* = 4.217, *p* < 0.0001; *FTH1*, *t* = 5.917, *p* < 0.0001). **(B)** SOCS1 and FTH1 protein levels in HNSCC were higher in HNSCC compared with those in normal tissues.

Given these findings that ferroptosis-related DEGs *SOCS1* and *FTH1* were independent prognostic factors in HNSCC, we next validated their higher levels using Oncomine and HPA data. The significantly higher *SOCS1* and *FTH1* mRNA levels in HNSCC compared with those in normal tissues were validated using the datasets GSE2379 (*SOCS1*, *t* = 3.569, *p* = 0.0010; *FTH1*, *t* = 3.168, *p* = 0.003), GSE3524 (*SOCS1*, *t* = 2.332, *p* = 0.0315; *FTH1*, *t* = 4.362, *p* = 0.0004), GSE6791 (*SOCS1*, *t* = 5.449, *p* < 0.0001; *FTH1*, *t* = 4.153, *p* = 0.0001), and PMID14729608 (*SOCS1*, *t* = 4.217, *p* < 0.0001; *FTH1*, *t* = 5.917, *p* < 0.0001) (Figure 3A). The protein levels of SOCS1 and FTH1 were correspondingly higher in HNSCC compared with those in normal tissues using HPA data (Figure 3B).

### *FTH1* mRNA Levels Positively Correlate With Lymph Node Metastasis

**FIGURE 4:**
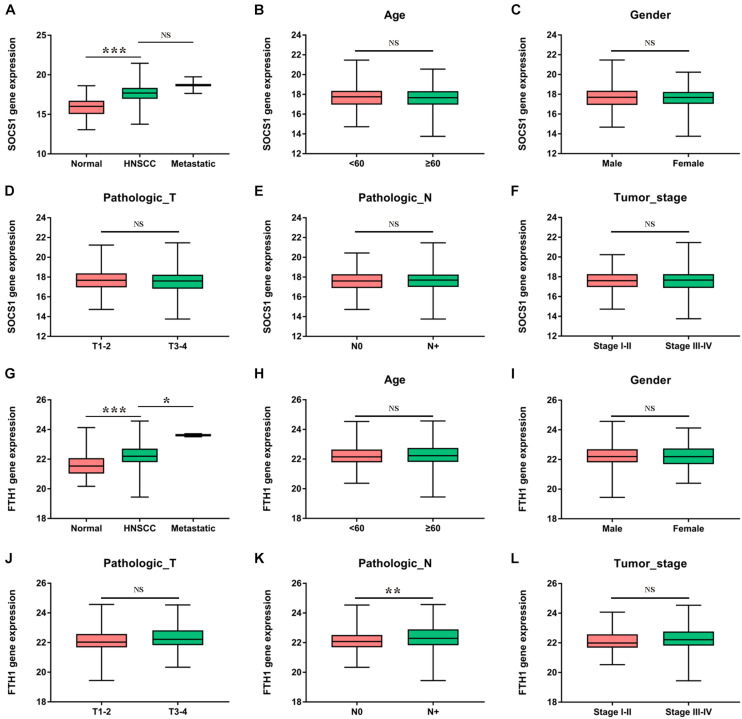
Clinical significance of *SOCS1* and *FTH1* gene expression in HNSCC. **(A)** *SOCS1* levels were significantly higher in HNSCC compared with those of normal tissues (*t* = 10.74, *p* < 0.001). **(B–F)** There was no statistically significant difference between *SOCS1* levels and age, sex, pathological T stage, pathological N stage, or tumor stage. **(G)** The levels of *FTH1* were significantly higher in HNSCC compared with those in normal tissues (*t* = 4.976, *p* < 0.001) and were significantly associated with metastatic sites (*t* = 2.569, *p* = 0.0105). **(H–L)** *FTH1* levels were significantly higher in HNSCC with node metastasis compared with HNSCC without such metastasis (*K*, *t* = 2.764, *p* = 0.0060), and there was no significant difference between *FTH1* levels and age, sex, pathological T stage, or tumor stage.

Furthermore, we analyzed the correlations between the mRNA levels of *SOCS1* and *FTH1* and clinical information (Figures 4A–L). *SOCS1* (Figure 4A, *t* = 10.74, *p* < 0.001) and *FTH1* (Figure 4G, *t* = 4.976, *p* < 0.001) mRNA levels were higher in HNSCC tissues compared with those in normal tissues. Furthermore, *FTH1* mRNA levels were higher in HNSCC with lymph node metastasis than without (Figure 4K, *t* = 2.764, *p* = 0.0060), consistent with the results of protein expression in our previous work (Hu et al., 2019).

### SOCS1 Expression Positively Correlates With M1 Macrophages, and *FTH1* Expression Positively Correlates With M2 Macrophages and TAMs

**FIGURE 5:**
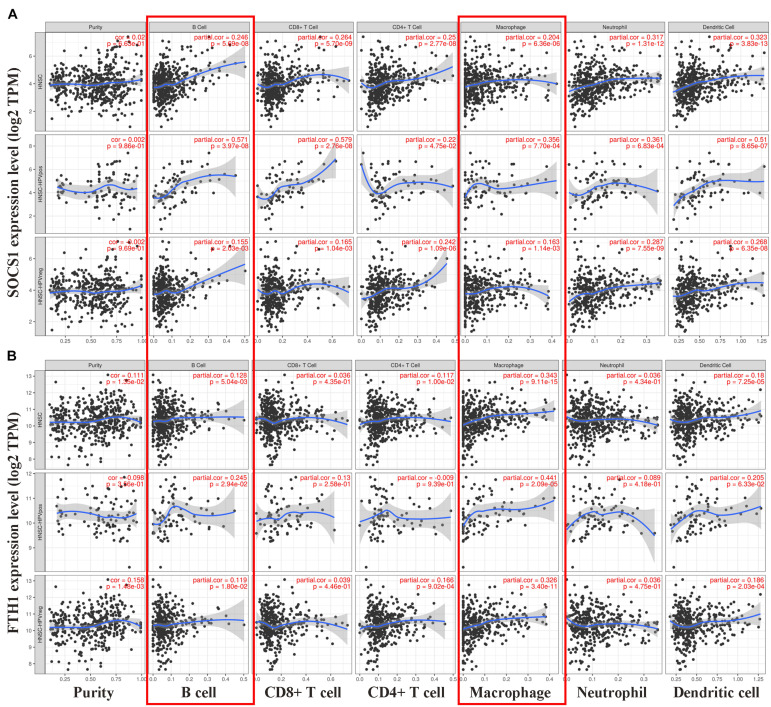
Correlations between *SOCS1* and *FTH1* expression and immune cell infiltration in HNSCC (TIMER). **(A)** *SOCS1* mRNA levels positively correlated with B cell and macrophage infiltration, independent of HPV status (*SOCS1* and B cells, *R* = 0.246, *p* = 5.69 × 10^–8^; *SOCS1* and macrophages, *R* = 0.204, *p* = 6.36 × 10^–6^). **(B)** *FTH1* mRNA levels positively correlated with B cell and macrophage infiltration, independent of HPV status (*FTH1* and B cells, *R* = 0.128, *p* = 5.04 × 10^–3^; *FTH1* and macrophages, *R* = 0.343, *p* = 9.11 × 10^–5^).

Finally, we conducted correlation analysis of the mRNA levels of *SOCS1* and *FTH1* and infiltrating immune cells. The TIMER data showed that the mRNA levels of *SOCS1* (Figure 5A) and *FTH1* (Figure 5B) were significantly associated with the infiltration of macrophages and B cells, regardless of HPV status (*SOCS1* and B cells, *R* = 0.246, *p* = 5.69 × 10^–8^; *SOCS1* and macrophages, *R* = 0.204, *p* = 6.36 × 10^–6^; *FTH1* and B cells, *R* = 0.128, *p* = 5.04 × 10^–3^; *FTH1* and macrophages, *R* = 0.343, *p* = 9.11 × 10^–5^).

**FIGURE 6:**
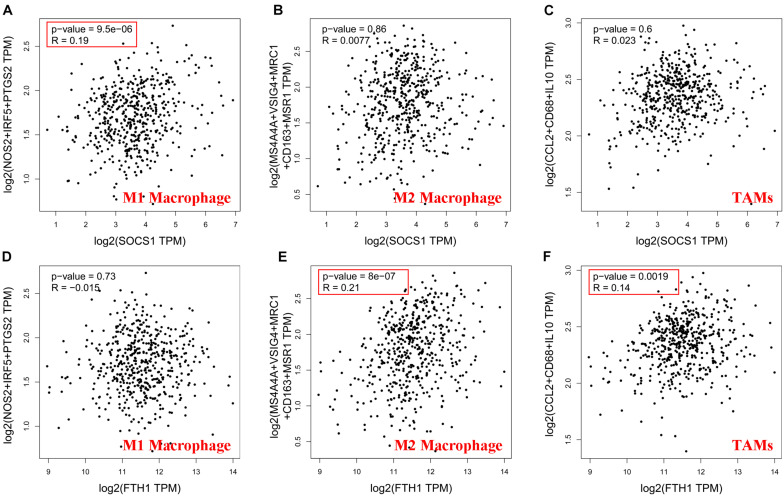
Significance of the correlations between *SOCS1* and *FTH1* expression and markers of macrophages (GEPIA2). **(A)** *SOCS1* and M1 (*R* = 0.19, *p* = 9.5 × 10^–6^). **(B)** *SOCS1* and M2 (*R* = 0.0077, *p* = 0.86). **(C)** *SOCS1* and TAMs (*R* = 0.023, *p* = 0.6). **(D)** *FTH1* and M1 (*R* = –0.015, *p* = 0.73). **(E)** *FTH1* and M2 (*R* = 0.21, *p* = 8 × 10^–7^). **(F)** *FTH1* and TAMs (*R* = 0.14, *p* = 1.9 × 10^–3^).

Then, GEPIA2 data were used to further analyze the correlation between *SOCS1* and *FTH1* levels and B-cell infiltration of tumors (Supplementary Figures 1A,B, *SOCS1* and B cells, *R* = 0.25, *p* = 1.2 × 10^–8^; *FTH1* and B cells, *R* = −0.12, *p* = 7.6 × 10^–3^) and macrophages through their immune cell signatures. The results of GEPIA2 verified the above correlations between *SOCS1* and *FTH1* levels and macrophage infiltration (Figure 6 and Supplementary Figures 1C–H). Furthermore, *SOCS1* expression was mainly related to M1 macrophages (*R* = 0.19, *p* = 9.5 × 10^–6^) (Figure 6A), while *FTH1* expression was significantly associated with M2 (*R* = 0.21, *p* = 8 × 10^–7^) (Figure 6E), TAMs (*R* = 0.14, *p* = 1.9 × 10^–3^) (Figure 6F), M2a (*R* = 0.14, *p* = 1.3 × 10^–3^) (Supplementary Figure 1F), M2b (*R* = 0.17, *p* = 1.1 × 10^–4^) (Supplementary Figure 1G), and M2c (*R* = 0.13, *p* = 3.2 × 10^–3^) (Supplementary Figure 1H).

**FIGURE 7:**
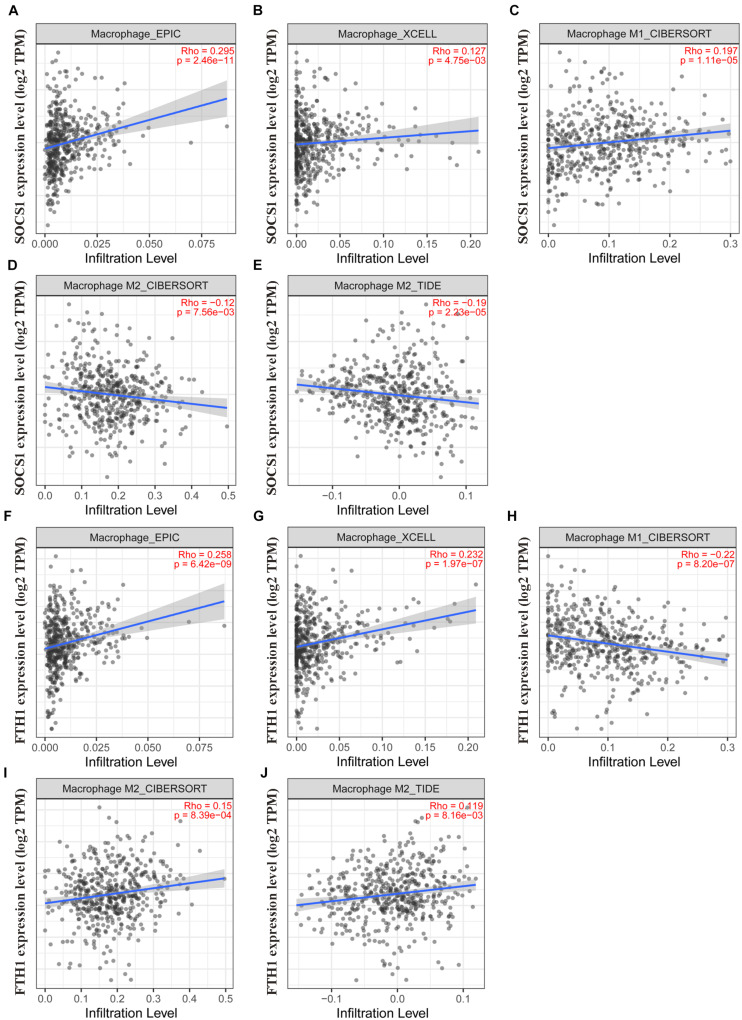
Validation of the significant correlations between *SOCS1* and *FTH1* expression and macrophages (TIMER2.0). **(A)** EPIC, *SOCS1*, and macrophages (*R* = 0.295, *p* = 2.46 × 10^–11^). **(B)** XCELL, *SOCS1*, and macrophages (*R* = 0.127, *p* = 4.75 × 10^–3^). **(C)** CIBERSORT, *SOCS1*, and M1 macrophages (*R* = 0.197, *p* = 1.11 × 10^–5^). **(D)** CIBERSORT, *SOCS1*, and M2 macrophages (*R* = –0.12, *p* = 7.56 × 10^–3^). **(E)** TIDE, *SOCS1*, and M2 macrophages (*R* = –0.19, *p* = 2.23 × 10^–5^). **(F)** EPIC, *FTH1*, and macrophages (*R* = 0.258, *p* = 6.42 × 10^–9^). **(G)** XCELL, *FTH1*, and macrophages (*R* = 0.232, *p* = 1.97 × 10^–7^). **(H)** CIBERSORT, *FTH1*, and M1 macrophages (*R* = –0.22, *p* = 8.2 × 10^–7^). **(I)** CIBERSORT, *FTH1*, and M2 macrophages (*R* = 0.15, *p* = 8.39 × 10^–4^). **(J)** TIDE, *FTH1*, and M2 macrophages (*R* = 0.119, *p* = 8.16 × 10^–3^).

Moreover, TIMER2.0 analysis further confirmed the correlations between *SOCS1* and *FTH1* levels and macrophage infiltration (A, EPIC, *SOCS1*, and macrophages, *Rho* = 0.295, *p* = 2.46 × 10^–11^; B, XCELL, *SOCS1*, and macrophages, *Rho* = 0.127, *p* = 4.75 × 10^–3^; C, CIBERSORT, *SOCS1*, and M1 macrophages, *Rho* = 0.197, *p* = 1.11 × 10^–5^; D, CIBERSORT, *SOCS1*, and M2 macrophages, *Rho* = −0.12, *p* = 7.56 × 10^–3^; E, TIDE, *SOCS1*, and M2 macrophages, *Rho* = −0.19, *p* = 2.23 × 10^–5^; F, EPIC, *FTH1*, and macrophages, *Rho* = 0.258, *p* = 6.42 × 10^–9^; G, XCELL, *FTH1*, and macrophages, *Rho* = 0.232, *p* = 1.97 × 10^–7^; H, CIBERSORT, *FTH1*, and M1 macrophages, *Rho* = 0.22, *p* = 8.2 × 10^–7^; I, CIBERSORT, *FTH1*, and M2 macrophages, *Rho* = 0.15, *p* = 8.39 × 10^–4^; J, TIDE, *FTH1*, and M2 macrophages *Rho* = 0.119, *p* = 8.16 × 10^–3^) (Figure 7).

Lastly, we investigated *SOCS1* and *FTH1* gene expression levels in various immune cells by CIBERSORT through GEPIA2021, the results revealed that M1 Macrophage has the highest median value of *SOCS1*, while M2 Macrophage has the highest median value of *FTH1* (Supplementary Figure 2).

## Discussion

Increasing recognition that ferroptosis plays complex roles in tumor biology fueled intense interest in its potential for developing novel cancer therapeutics (Lei et al., 2020; Chen et al., 2021). The induction of ferroptosis requires iron accumulation, lipid peroxidation, and membrane damage (Zheng and Conrad, 2020). Furthermore, a large quantity of iron is required to support the rapid proliferation cancer cells in most solid tumors (Torti and Torti, 2013; Manz et al., 2016). For example, we previously found that HNSCC tissue accumulates iron, particularly in metastatic tissue (Hu et al., 2019). Moreover, cancer cells maintain high metabolic activity and a higher load of reactive oxygen species (Jiang et al., 2021). Therefore, we believe that it is reasonable to assume that tumor cells are susceptible to ferroptosis. Furthermore, tumor cells may upregulate the expression of ferroptosis suppressors to counteract the driver to inhibit the antitumor function of ferroptosis (Rojo et al., 2018).

Here we show that the levels of ferroptosis driver *SOCS1* (Saint-Germain et al., 2017) and suppressor *FTH1* (Du et al., 2019) positively correlated among each other and were upregulated in HNSCC compared with their levels in normal tissues. The *SOCS1* gene, which resides on human chromosome 16p13.3, encodes a 211 amino-acid polypeptide chain (Yandava et al., 1999). The main function of *SOCS1* is to suppress cell signaling and promote ubiquitination through recruiting E3 ubiquitin ligases (Ying et al., 2019). *SOCS1* induces ferroptosis through p53 target genes and downregulation of the expression of SLC7A11 (Saint-Germain et al., 2017). Thus, *SOCS1* functions as a tumor suppressor, and the inhibition of this function may promote cancer progression or relapse. For example, a study of 83 patients with esophageal cancer found that exosome-associated miR-19b-3p promotes tumor progression by inhibiting *SOCS1* expression (Deng et al., 2021). Moreover, loss of SPTBN1 expression induces liver cancer through downregulation of *SOCS1* expression as well (Lin et al., 2021). In nasopharyngeal carcinoma, LINC00669 protects *SOCS1* from ubiquitinating STAT1, which promotes cancer cell proliferation and invasion (Qing et al., 2020). Here we show that *SOCS1* was differentially expressed at higher levels in HNSCC and served as a significant and independent prognostic factor for HNSCC, consistent with its role in hepatocellular carcinoma (Khan et al., 2020).

*FTH1*, the functional subunit of the major iron storage protein ferritin, possesses ferroxidase activity and efficiently reduces the toxicity of Fe^2+^ (Salatino et al., 2019). Furthermore, *FTH1* protect cancer cells from ferroptosis (Sun et al., 2016; Du et al., 2019). Here, we show that *FTH1* was expressed at higher levels in HNSCC compared with those of normal tissues, which is consistent with the findings of our previous study (Hu et al., 2019). Furthermore, *FTH1* served as an independent prognostic factor of poorer prognosis, in contrast to *SOCS1*.

The multiple roles of ferroptosis in tumor immunity is attracting intense interest, which mainly focuses on CD8+ T cells that induce ferroptosis of cancer cells through secreting interferon gamma (Wang et al., 2019). In contrast, CD36-mediated ferroptosis impairs antitumor activity through dampening the functions of CD8+ T cells (Ma et al., 2021). The macrophage in the tumor immune microenvironment may exert dual influences on tumors depending on their phenotypic polarization (Wang et al., 2020; Maller et al., 2021). For example, in the M1–M2 macrophage polarization system, macrophages are typically divided into an antitumor M1, classically activated subtype, in the presence of high levels of TNF, NOS2, or MHC2. The alternative subtype, protumor M2 macrophages, are activated in the presence of high levels of ARG1, IL-10, CD163, CD204, or CD206 (Mantovani et al., 2002; Mantovani and Locati, 2013). Additionally, based on the different cytokine expression profiles, M2 could be further subdivided into M2a, M2b, M2c, and M2d (aka. tumor-associated macrophage, TAM), all of them share the immunosuppressive functions (Avila-Ponce De León et al., 2021). Furthermore, in tumors the microenvironment tends to induce M2-like TAMs (Shan et al., 2020).

To our knowledge, few studies focus on the role of ferroptosis in tumor infiltrating macrophages. Our previous analysis of diverse cancers found that in HNSCC, *FTH1* expression positively correlates with infiltration by macrophages of most solid tumors (Hu et al., 2021). Nevertheless, we were unable to identify the subtypes. Here we show that *FTH1* expression positively correlated with M2-macrophage infiltration and that in contrast, the ferroptosis driver *SOCS1* was mainly associated with M1-macrophage infiltration. We therefore hypothesize that the balance between the driver *SOCS1* and suppressor *FTH1* influence macrophage polarization through the regulation of ferroptosis.

An increasing number of studies focus on therapeutic targeting of TAMs, which mainly include depletion and repolarization of macrophages (DeNardo and Ruffell, 2019). The main methods used to deplete TAMs include the inhibition of CCL2–CCR2 signaling or the activity of the CSF1–CSF1R axis, both of which reduce the numbers of TAMs in tumor sites and improve the efficacy of immunotherapy (Kalbasi et al., 2017; Peranzoni et al., 2018; Wang et al., 2018). However, the macrophage is the primary phagocyte and antigen-presenting cell in the TME, and the depletion of TAMs inhibit their latent immune stimulatory function. Thus, repolarization of TAMs from the M2 to the M1 phenotype may serve as a more efficacious approach to improving the efficacy of immunotherapy (Goossens et al., 2019). We speculate that this may be achieved through the induction of ferroptosis in HNSCC through increasing the expression of *SOCS1* or decreasing that of *FTH1*.

In summary, we show here that the ferroptosis driver *SOCS1* and suppressor *FTH1* served as independent prognostic factors that independently correlate with M1 and M2 macrophage infiltration in HNSCC, suggesting that inducing ferroptosis directly influences the infiltration of M1–M2 macrophages. The targeting of ferroptosis-immunomodulation may therefore serve as a strategy to enhance the activity of immunotherapy.

## Data Availability Statement

Publicly available datasets were analyzed in this study. This data can be found here: https://genomecancer.ucsc.edu/; https://xenabrowser.net/; http://www.zhounan.org/ferrdb/; https://string -db.org/; http://www.genemania.org; http://gepia.cancer-pku.cn/index.html; http://kmplot.com/analysis/; https://www.oncomine. org; https://www.proteinatlas.org/; https://cistrome.shinyapps.io/timer/; http://gepia2.cancer-pku.cn/#index; http://timer.comp-genomics.org/; http://gepia2021.cancer-pku.cn/; and http://cis.hku.hk/TISIDB/.

## Author Contributions

Z-WH, WS, and W-PW conceived and designed the study. Y-HW, X-LZ, and LC downloaded the data, while Z-WH and R-QM analyzed the data. Z-WH wrote the manuscript. All authors read and approved the final manuscript.

## Conflict of Interest

The authors declare that the research was conducted in the absence of any commercial or financial relationships that could be construed as a potential conflict of interest.

## Publisher’s Note

All claims expressed in this article are solely those of the authors and do not necessarily represent those of their affiliated organizations, or those of the publisher, the editors and the reviewers. Any product that may be evaluated in this article, or claim that may be made by its manufacturer, is not guaranteed or endorsed by the publisher.

## Footnotes

1
https://genomecancer.ucsc.edu/

2
https://xenabrowser.net/

3
http://www.zhounan.org/ferrdb/

4
https://string-db.org/

5
http://www.genemania.org

6
http://gepia.cancer-pku.cn/index.html

7
http://kmplot.com/analysis/

8
https://www.oncomine.org

9
https://www.proteinatlas.org/

10
https://cistrome.shinyapps.io/timer/

11
http://gepia2.cancer-pku.cn/#index

12
http://timer.comp-genomics.org/

13
http://gepia2021.cancer-pku.cn/

## Supplementary Material

Supplementary Figure 1 Correlations between *SOCS1* and *FTH1* gene expressions and markers of B cell, M2a, M2b, and M2c (GEPIA2). **(A)** *SOCS1* and B cell (*R* = 0.25, *p* = 1.2 × 10^–8^). **(B)** *FTH1* and B cell (*R* = −0.12, *p* = 7.6 × 10^–3^). **(C)** *SOCS1* and M2a (*R* = −0.011, *p* = 0.8). **(D)** *SOCS1* and M2b (*R* = 0.005, *p* = 0.91). **(E)** *SOCS1* and M2c (*R* = 0.08, *p* = 6.8 × 10^–2^). **(F)** *FTH1* and M2a (*R* = 0.14, *p* = 1.3 × 10^–3^). **(G)** *FTH1* and M2b (*R* = 0.17, *p* = 1.1 × 10^–4^). **(H)** *FTH1* and M2c (*R* = 0.13, *p* = 3.2 × 10^–3^).

Supplementary Figure 2 *SOCS1* and *FTH1* gene expressions in various immune cells by CIBERSORT (GEPIA2021). M1 Macrophage has the highest median value of *SOCS1* (median value 3.3212), while M2 Macrophage has the highest median value of *FTH1* (median value 5.4181).

Supplementary Table 1 A list of ferroptosis related genes from the FerrDb database.

Supplementary Table 2 Correlations of expressions between each two genes of the 18 selected ferroptosis related genes.

Supplementary Table 3 Annotation of ferroptosis-related differentially expressed proteins and their co-expression scores (STRING analysis).
