# Supplementary figures and images for: Ferroptosis Driver SOCS1 and Suppressor FTH1 Independently Correlate With M1 and M2 Macrophage Infiltration in Head and Neck Squamous Cell Carcinoma
Source: Front Cell Dev Biol. 2021 Aug 30;9:727762. doi: 10.3389/fcell.2021.727762 (PMC8437260; doi:10.3389/fcell.2021.727762)

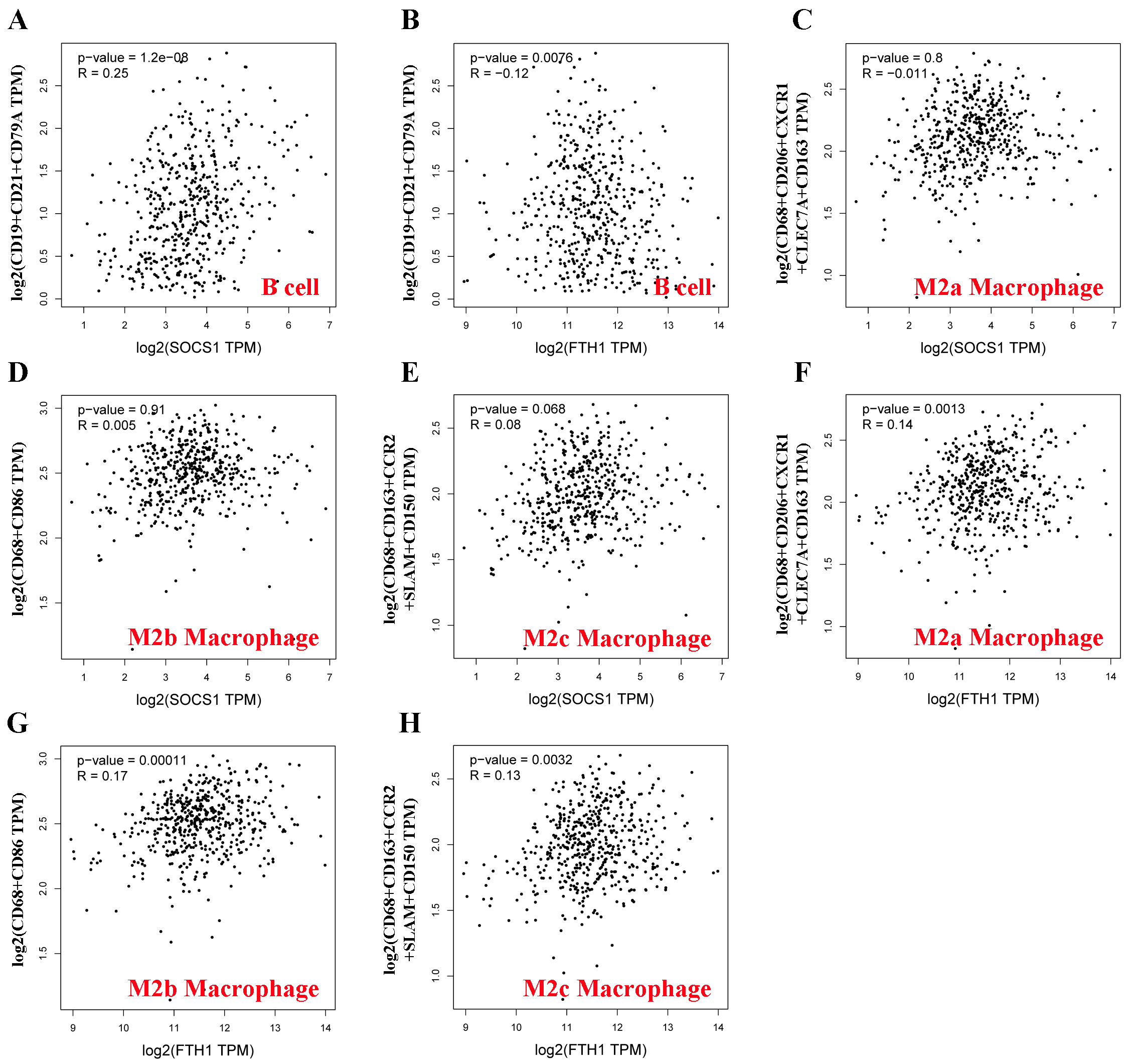

Supplement: Supplementary Figure 1 — Correlations between SOCS1 and FTH1 gene expressions and markers of B cell, M2a, M2b, and M2c (GEPIA2). (A) SOCS1 and B cell (R = 0.25, p = 1.2 × 10^–8^). (B) FTH1 and B cell (R = −0.12, p = 7.6 × 10^–3^). (C) SOCS1 and M2a (R = −0.011, p = 0.8). (D) SOCS1 and M2b (R = 0.005, p = 0.91). (E) SOCS1 and M2c (R = 0.08, p = 6.8 × 10^–2^). (F) FTH1 and M2a (R = 0.14, p = 1.3 × 10^–3^). (G) FTH1 and M2b (R = 0.17, p = 1.1 × 10^–4^). (H) FTH1 and M2c (R = 0.13, p = 3.2 × 10^–3^). [file Image_1.TIF]

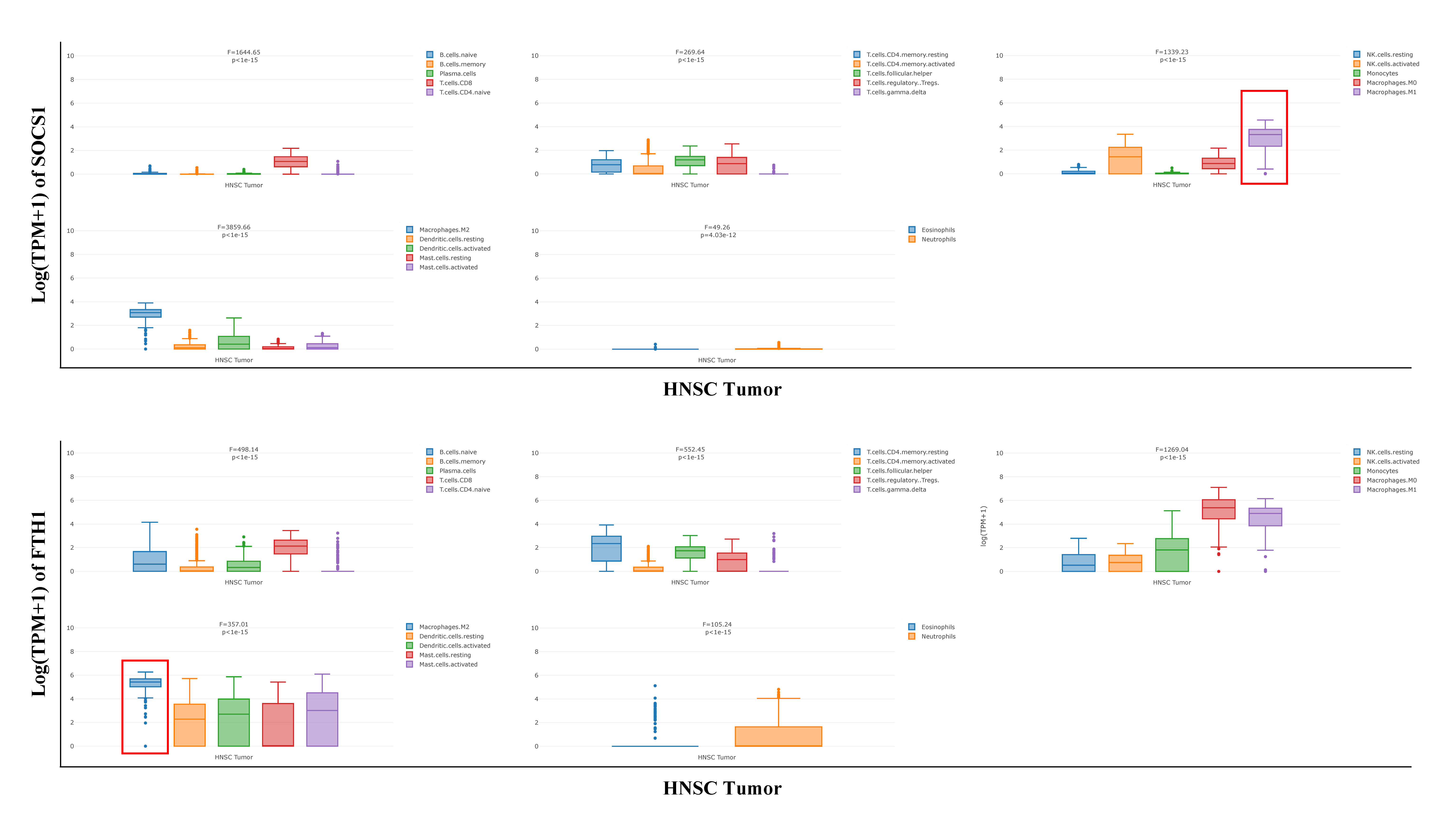

Supplement: Supplementary Figure 2 — SOCS1 and FTH1 gene expressions in various immune cells by CIBERSORT (GEPIA2021). M1 Macrophage has the highest median value of SOCS1 (median value 3.3212), while M2 Macrophage has the highest median value of FTH1 (median value 5.4181). [file Image_2.TIF]

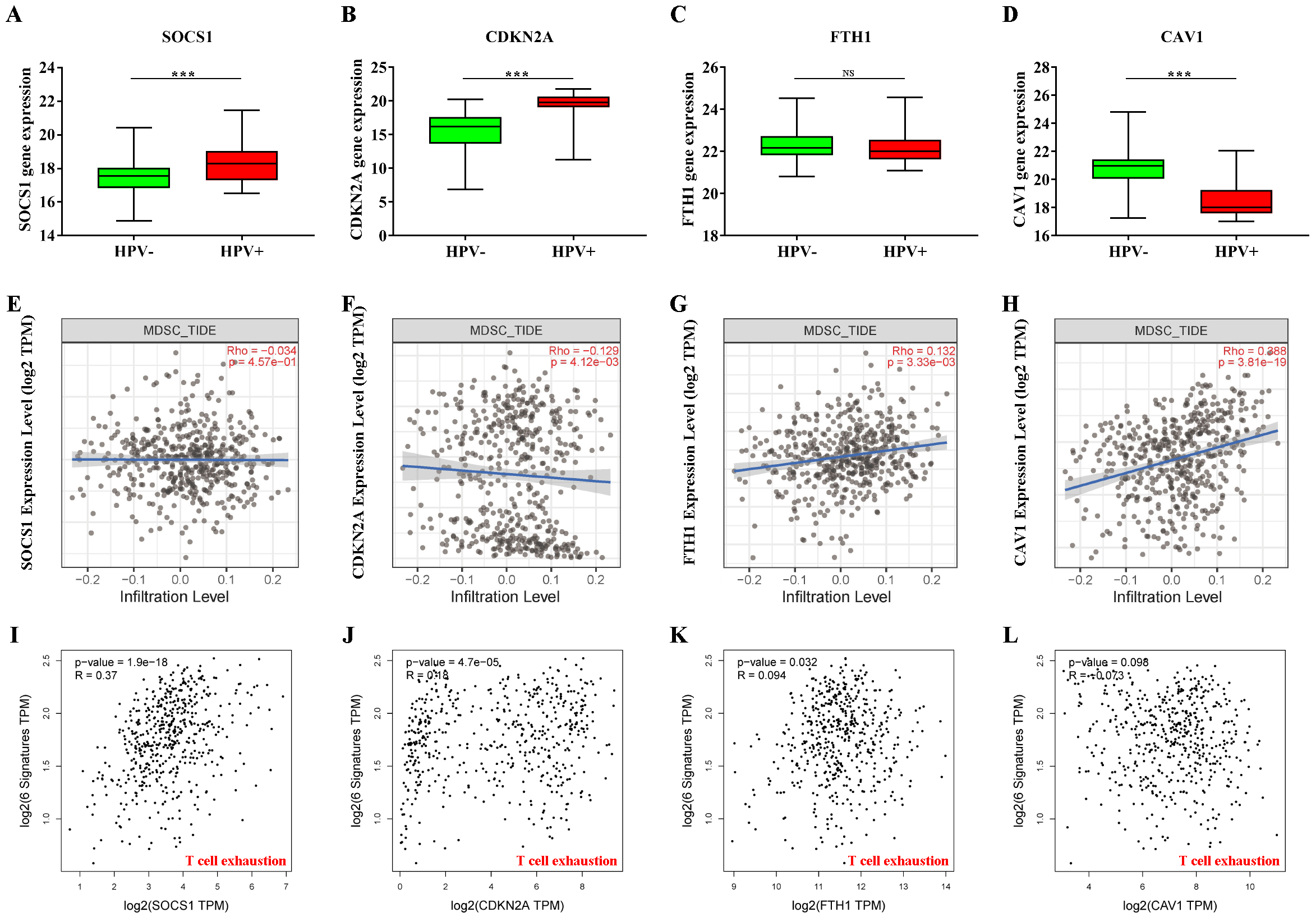

Supplement: Supplementary file 3 [file Image_3.TIF]

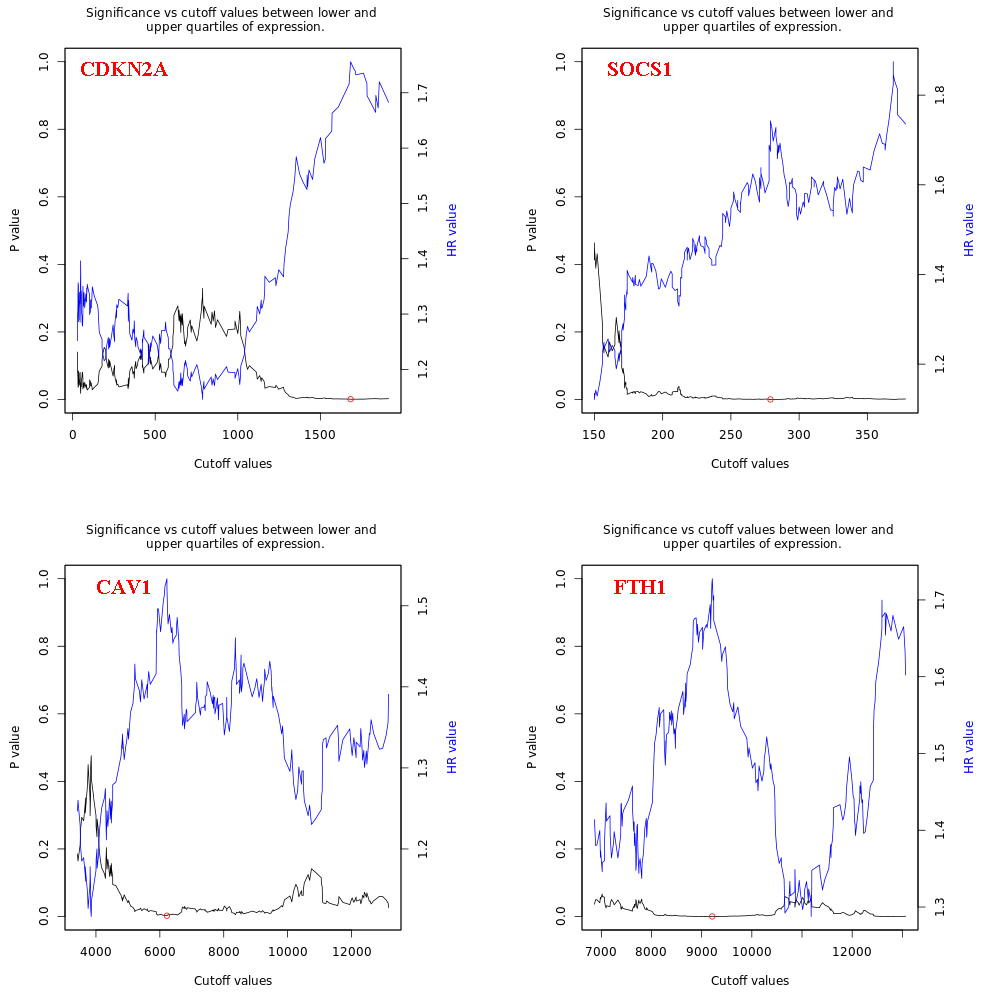

Supplement: Supplementary file 4 [file Image_4.TIF]
